# Laser Dissimilar Welding of AISI 430F and AISI 304 Stainless Steels

**DOI:** 10.3390/ma13204540

**Published:** 2020-10-13

**Authors:** Krzysztof Pańcikiewicz, Aleksandra Świerczyńska, Paulina Hućko, Marek Tumidajewicz

**Affiliations:** 1 Faculty of Metals Engineering and Industrial Computer Science, AGH University of Science and Technology, al. Adama Mickiewicza 30, 30-059 Kraków, Poland; paulina.hucko@gmail.com; 2 Faculty of Mechanical Engineering, Gdańsk University of Technology, Gabriela Narutowicza 11/12, 80-233 Gdańsk, Poland; aleksandra.swierczynska@pg.edu.pl; 3 MOSTOSTAL KRAKÓW S.A., Ujastek 7, 30-969 Kraków, Poland; 4 RaQun Sp. z o.o., Fabryczna 4, 38-300 Gorlice, Poland; m.tumidajewicz@raqun.pl

**Keywords:** laser welding, austenitic stainless steel, martensitic stainless steel, dissimilar welded joint, microstructure, sulfide inclusions

## Abstract

A dissimilar autogenous laser welded joint of AISI 430F (X12CrMoS17) martensitic stainless steel and AISI 304 (X5CrNi18-10) austenitic stainless steel was manufactured. The welded joint was examined by non-destructive visual testing and destructive testing by macro- and microscopic examination and hardness measurements. With reference to the ISO 13919-1 standard the welded joint was characterized by C level, due to the gas pores detected. Microscopic observations of AISI 430F steel revealed a mixture of ferrite and carbides with many type II sulfide inclusions. Detailed analysis showed that they were Cr-rich manganese sulfides. AISI 304 steel was characterized by the expected austenitic microstructure with banded δ-ferrite. Martensitic microstructure with fine, globular sulfide inclusions was observed in the weld metal. The hardness in the heat-affected zone was increased in the martensitic steel in relation to the base metal and decreased in the austenitic steel. The hardness range in the weld metal, caused by chemical inhomogeneity, was 184–416 HV0.3.

## 1. Introduction

The development of technology is aimed at reducing production and operating costs, increasing the safety of use and improving ecology in the context of the development of materials. This opens the possibility of using new materials, developing the existing ones and modifying their properties [1]. Particularly in the area of welded joints, especially dissimilar joints, there are many opportunities due to the multitude of materials, processes and additional treatments that can significantly affect the final product.

AISI 430F (X12CrMoS17) is a stainless steel with the addition of sulfur typically existing in the form of MnS inclusions, which improves the machinability of this material. Despite the high chromium content, the addition of sulfur lowers the resistance to crevice and pitting corrosion. Depending on the type of treatment, it has a ferritic or ferritic-martensitic structure [2]. This steel is not recommended for use in marine environments and highly oxidizing chemical environments. It is used in the production of screws, spindles, nuts, medical instruments, and in the automotive and transport industry. Due to its applications, this steel can be subjected to plasma nitriding or nitrocarburizing to increase hardness, corrosion, tribocorrosion and wear resistance [3,4,5].

AISI 304 (X5CrNi18-10) steel is a very popular grade of austenitic stainless steel, with good corrosion resistance. It is used in the construction, automotive, food, and chemical industries, for decorative purposes and kitchen equipment, as well as an element of electronic equipment [6]. There have been many studies describing the corrosion resistance, mechanical and functional properties of this steel [7,8]. Currently, many studies have focused on possible ways to improve the properties of AISI 304 steel [9,10,11].

The weldability of steel depends primarily on its chemical composition, structure, mechanical and physicochemical properties. Therefore, dissimilar steels usually require special care to be able to join them [12,13,14]. The basic difficulties include differences in the values of thermal expansion coefficients, melting points, mechanical properties, electrochemical potential as well as the formation of intermetallic compounds [15]. Additional difficulties may arise from the need to meet different heat treatment requirements for each of the welded materials. Dissimilar joints are characterized by a non-linear change of properties and often a very unfavorable segregation of elements, which may eliminate them from service. In addition, dissimilar joining requires extensive technical knowledge, appropriate equipment and careful selection of consumables. However, these inconveniences and risks are taken due to the many benefits that arise from combining two very different materials, reducing both production and operating costs and the improving mechanical properties of the joints [16,17]. In order for such a joining to be carried out without unacceptable imperfections, it is necessary to thoroughly understand the technologies and weldability of materials.

AISI 430F steel is usually regarded as difficult to weld due to, among other factors, hydrogen-induced cold cracking, lack of weld ductility, intergranular corrosion, deterioration of toughness and corrosion resistance. In order to carry out the welding it is necessary to use pre-heating at 150–200 °C, which reduces diffusible hydrogen content and welding stresses. Additionally, post-weld annealing at 790–815 °C can be performed. To reduce the risk of intergranular corrosion, chrome depletion at grain boundaries should be decreased by the use of low heat input value. Despite so many difficulties, the literature shows that there have been few effective attempts to weld this steel grade, mainly to make dissimilar joints in such applications as pressure vessels or fuel injectors. Khan et al. [18] investigated laser beam welding of dissimilar AISI 430F and AISI 440C stainless steels and the effects of laser welding parameters and heat input on weld bead geometry. They obtained qualitatively sound joints by finding appropriate welding parameters to avoid micro-crack formation. Romoli et al. [19] made a dissimilar AISI 440C with AISI 430F laser welded joint and found an empirical relationship between the shear strength of the weld and the configuration adopted during experiments. They observed that higher susceptibility to surface crack formation generated on the boundary of the weld seam with AISI 440C is connected with a higher martensitic volume fraction. 

AISI 304 steel is considered to be easily weldable, with the use of specific technological procedures, such as ensuring that the interpass temperature does not exceed 200 °C. However, it should be remembered that the heat introduced into the material during welding makes it more sensitive to intergranular corrosion and solidification cracking. Pankaj et al. [20] investigated the possibility of laser welding of AISI 304 steel with low carbon steel and found a correlation between heat input and the properties of the joints. Rogalski et al. [21] welded 304L steel with Incoloy 800HT and conducted an analysis of the properties and structure of the joint, confirming that the TIG method can be used to join these materials even with an unfavorable design resulting from the working conditions of the joint. On the other hand, Pańcikiewicz et al. [22] indicated in their work that although austenitic steels are treated as easily weldable, they cannot be ignored and potentially dangerous imperfections should be identified using both traditional and new test methods. At the same time, Kurc-Lisiecka et al. [23] obtained the sound joints during laser welding of AISI 304 steel in a wide range of welding parameters, confirming the good weldability of this material, even when using a concentrated heat source. Welding of AISI 304 austenitic steel with AISI 430 ferritic steel was performed by Wang et al. [24]. They used the gas tungsten arc welding process, assessed the structure and corrosion resistance of welded joints and proved that it is possible to obtain the correct joint between these steels.

Laser welding by concentrating the heat source provides many opportunities compared to the conventional arc welding: simplicity of automation, very narrow HAZ, slight material deformation, deep penetration without the need for beveling and the use of filler metal [25,26]. These features make laser technologies are constantly evolving so that the expensive equipment, which is necessary in this method, is becoming more accessible and at the same time the process simulation programs are more and more accurate [27,28]. In addition, laser welding was initially considered unsuitable for difficult-to-weld materials and carried a high risk due to the concentrated heat source. A lot of research has been done on the possibility to use laser welding for joining various materials and show that this method makes it possible to obtain high quality welded joints using the wide range of conditions and parameters [29,30,31].

Each of the previously discussed steel grades can cause some problems during and after welding. According to the literature, each of the steel grades was subjected to laser welding, but there are no data on the possibility of laser beam welding of AISI 430F steel with AISI 304 steel. The purpose of this work was to analyze the possibility of making the overlap autogenous welded joint with the use of laser beam welding and to show the mechanism of structure formation in this welded joint. This goal was achieved by metallographic macro- and microscopic observations, EDS analysis, simulations and hardness measurements.

## 2. Materials and Methods

**Figure 1:**
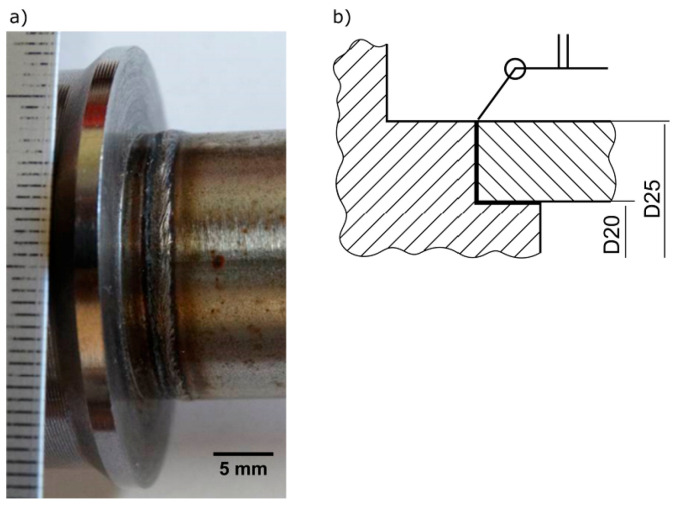
The welded joint: (**a**) general view, (**b**) design.

The overlap joint of AISI 430F drill rod and AISI 304 tube (Ø25 × 2.5 mm) was welded by the 521 laser beam welding (LBW) process without a filler metal. A general view and the design of the welded joint are presented in Figure 1. This joint, a part of a specialized drill produced by RaQun Sp. z o.o. (Gorlice, Poland) for the mining industry, was made during the start of development and first testing of the welding procedure qualification. 

Visual testing was performed according to European Standards ISO 17637 and EN 13018 by direct method in intense lighting ~600 lx with universal welding gauge, with resolution 0.05 mm. Chemical composition of base metals was measured by optical emission spectroscopy (OES) using a Foundry Master-WAS Spectrometer (Hitachi, Tokyo, Japan). Macro- and microscopic examinations were performed on two cross sections after mechanical grinding, polishing and two-step etching: chemical in Kalling’s reagent (5 g CuCl_2_ + 100 mL HCl + 100 mL C_2_H_5_OH) and electrolytic in 10% CrO_3_ water solution. Base metals, heat-affected zones and weld metal were examined. Microstructure was characterized using a light microscope (Leica, Wetzlar, Germany) and scanning electron microscope (SEM) (Japan Electron Optics Laboratory Co., Ltd., Tokyo, Japan) with X-ray energy-dispersive spectroscopy (EDS) (Japan Electron Optics Laboratory Co., Ltd., Tokyo, Japan). The depth of EDS analysis was assessed by CASINO v3.3.0.4 software (Université de Sherbrooke, Sherbrooke, QC, Canada). Thermodynamic analyses were performed by Thermo-Calc 2020a (Thermo-Calc Software AB, Solna, Sweden) with the TC-FE7 database (Thermo-Calc Software AB, Solna, Sweden). Hardness measurements were performed by the Vickers method (2.942 N) on a Tukon 2500 universal hardness tester (Wilson, NY, USA) in 8 rows of 25 indentations on the cross section of the joint. The distance between the indentations was 0.2 mm and indentation time was 5 s.

## 3. Results and Discussion

Chemical compositions of base metals, verified by the OES, and weld metal, verified by the EDS, are presented in Table 1. The compositions comply with the requirements of product standards. The main alloying elements in AISI 430F steel were 15.5% chromium for corrosion resistance and almost 0.5% of sulfur for machinability in automatic machining processes. AISI 304 steel contains 18% chromium and 8% nickel to provide an austenitic structure with δ-ferrite at ambient temperature.

**Figure 2:**
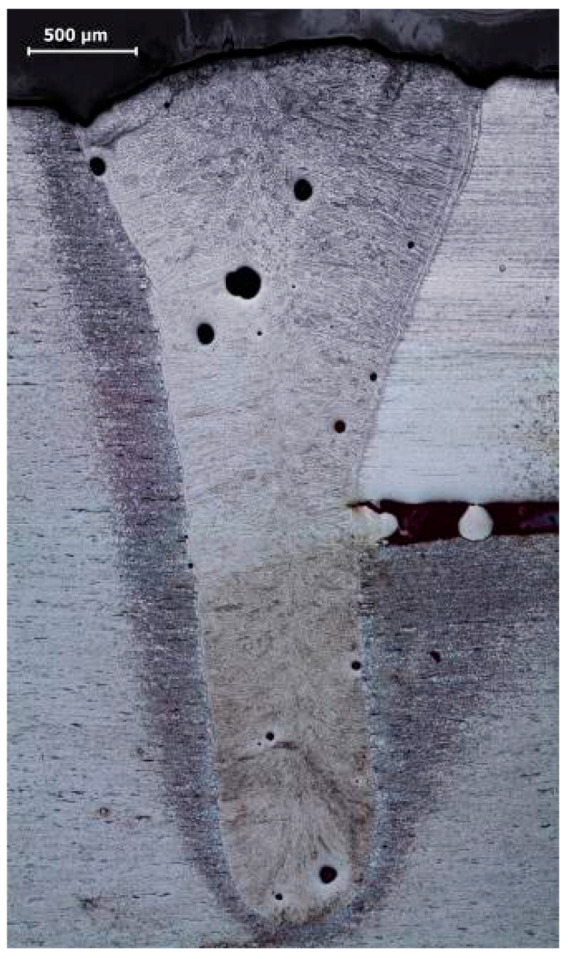
Macrostructure of the welded joint.

The butt welded joint was B quality level (acc. to EN ISO 13919-1) in visual testing (VT) and was C quality level in macroscopic examination. Full penetration and a few gas pores were observed (Figure 2). The largest pore was 0.16 mm in diameter and all the pores occupied 1.2% of the weld area (Table 2). For C quality level, the maximum dimension of the pore (with a tube thickness of 2.5 mm) was 1 mm and the maximum dimension of the area of the imperfection related to the projected area was 2%.

**Figure 3:**
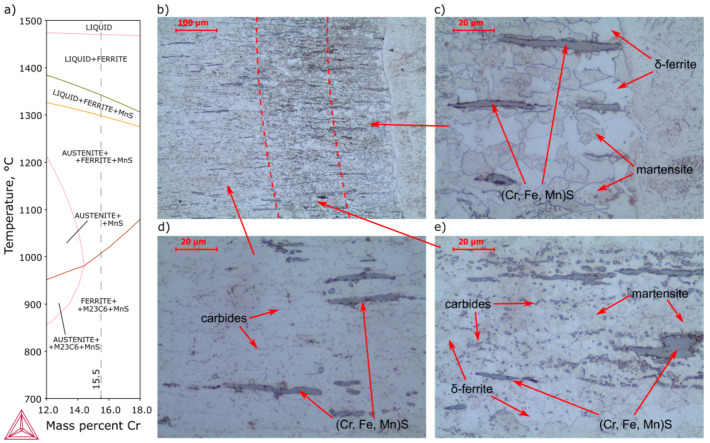
Microstructure of AISI 430F (X12CrMoS17) steel heat-affected zone: (**a**) pseudobinary phase diagram of 0.17C-0.48Si-0.8Mn-0.028P-0.497S-0.26Mo-0.33Ni-0.12Cu-0.05V steel, (**b**) general view, (**c**) coarse-grain heat-affected zone, (**d**) base metal, (**e**) intercritical heat-affected zone (Kalling’s and CrO_3_ etched).

**Figure 4:**
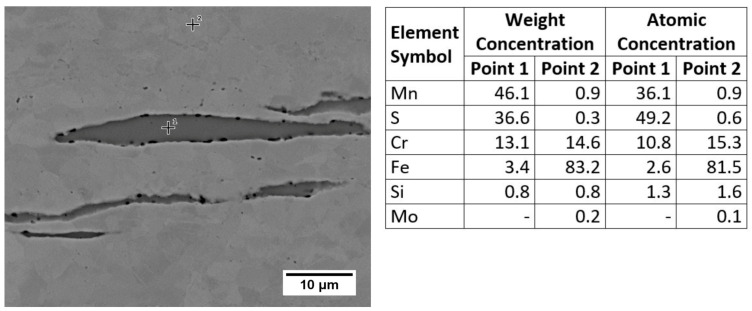
SEM microstructure and EDS point analysis of AISI 430F (X12CrMoS17) base metal (polished).

Figure 3 presents the microstructure on the AISI 430F steel side of the joint. Base metal (Figure 3d) was characterized by a mixture of ferrite and carbides, which indicates a soft annealing or a high tempering delivery condition. This is a particularly favorable condition for machining due to the avoidance of a martensitic structure and low hardness. Gray inclusions elongated in the rolling direction, characteristic of manganese sulfides, were also observed (Figure 3d). The shape of the inclusions suggests that they are type II sulfides, acc. to Sims’s classification [32]. EDS analysis confirmed the high content of manganese and sulfur, and additionally the inclusion was enriched by chromium (Figure 4), as indicated by a similar chromium content in the inclusion and the matrix. In high chromium steels it is possible to enrich the inclusions with this element. Considering also the small amount of iron, pyrrhotite with the formula (Cr, Fe, Mn)S can be formed [33,34,35]. Atomic concentration analysis indicates a 3:1 ratio between iron+manganese and chromium, and a 1:1 ratio between metallic elements and sulfur; thus, the observed inclusions were [(Fe,Mn)_3_Cr]S.

In the heat-affected zone, Figure 3b, intercritical HAZ (ICHAZ, heated to the δ + γ + MnS range) and coarse-grain HAZ (CGHAZ, heated much above A3) were observed. In ICHAZ an incomplete normalized microstructure with fine and medium grains and more carbides than base metal (Figure 3e) and in CGHAZ an overheated microstructure with coarser grains than in other zones were observed (Figure 3c). Due to the presence of a higher content of ferrite stabilizers (particularly chromium), martensite with δ-ferrite was observed, clearly visible in CGHAZ (Figure 3c). With increasing chromium content, the γ-field on the phase diagram contracts to a small region, and with increasing carbon content, initially two-phase (δ + γ)-field expands. Accelerated cooling in the two-phase field, in the absence or narrow range of homogeneous austenite, leads to the transformation of the austenite into martensite and some δ-ferrite remaining (Figure 3a). Unfortunately, a negative effect on mechanical properties is observed – ductile-brittle transition temperature increases and tensile strength decreases with increase δ-ferrite in volume fraction [36,37].

**Figure 5:**
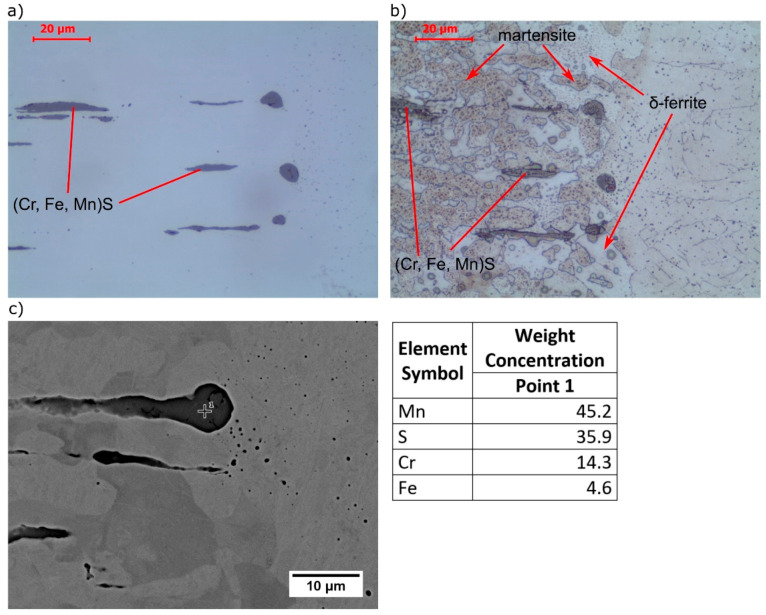
Microstructure near fusion line on the AISI 430F (X12CrMoS17) base metal side: (**a**) microstructure (polished), (**b**) microstructure (Kalling’s etched), (**c**) SEM microstructure and EDS point analysis (polished).

**Figure 6:**
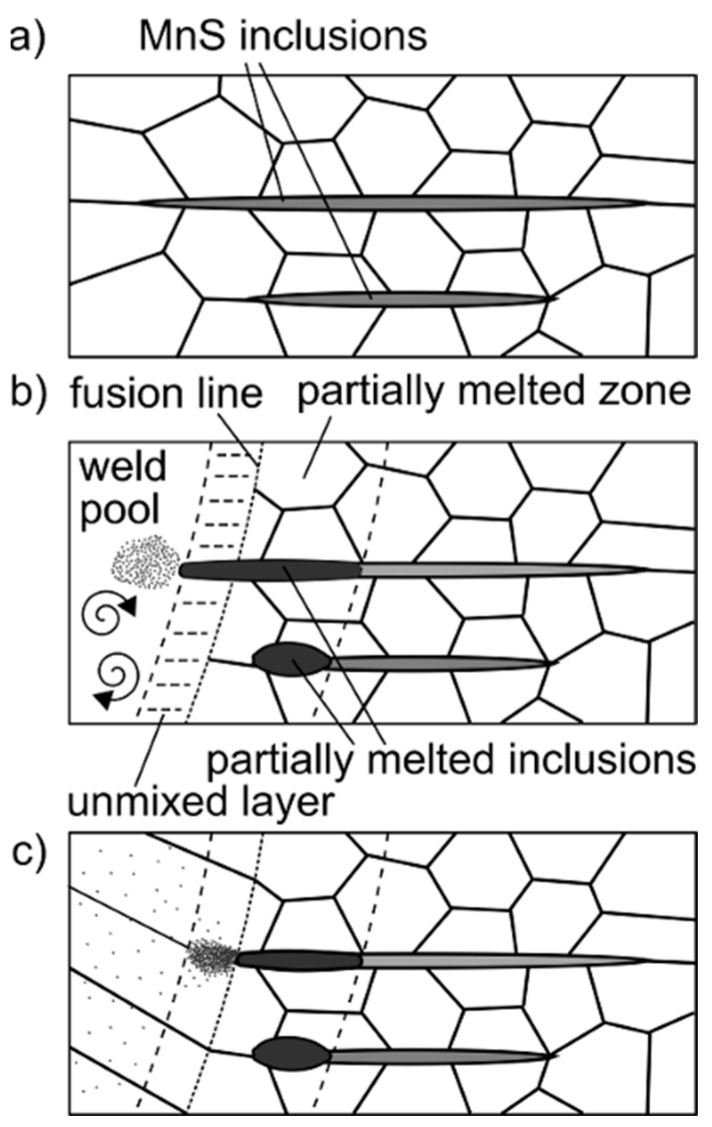
Constitutional liquation mechanism of sulfide in partially melted zone. (**a**) before welding; (**b**) during welding; (**c**) after welding.

Near the fusion line, on both sides, the shape of the inclusions was changed from elongated to spherical, which was caused by high temperature from laser beam welding (Figure 5). The mechanism is shown in Figure 6. 

**Figure 7:**
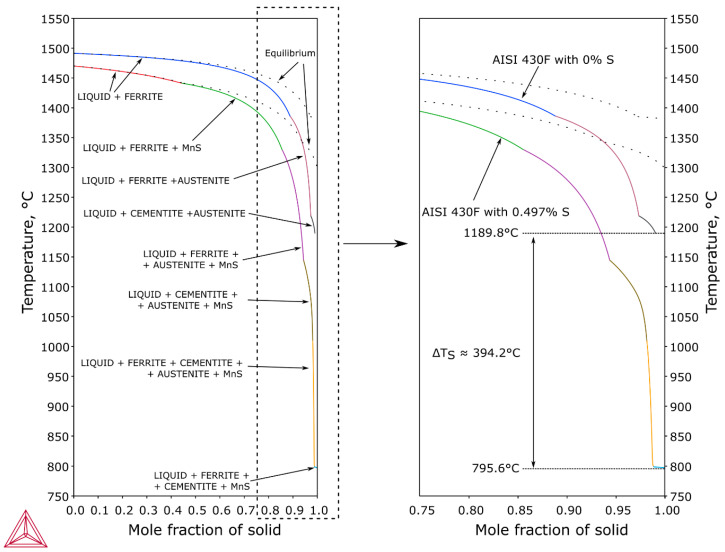
AISI 430F (X12CrMoS17) steel solidification (Scheil Simulation).

Sulfides, which were in the high-temperature HAZ, melted with the formation of a liquid film as a result of constitutional liquation. Part of the sulfur-rich liquid was located at grain boundaries in the partially melted zone, while part was located in the weld. At the fusion line, sulfur-rich liquid was not mixed with the weld metal due to the presence of an unmixed zone near the fusion line (Nernst layer). During cooling, the areas with a higher melting point (poorer in impurity) were first crystallized. Figure 7 shows a Scheil Simulation for AISI 430F steel solidification, when the chemical composition corresponds to the tested steel (sulfur mass content was 0.497%) and when the sulfur content was 0%. The solidification temperature of steel with 0.497%S was lower than that of the sulfur-free steel by 394.2 deg. Sulfur-rich liquid is trapped at the grain boundaries and crystallizes in the form of eutectics.

**Figure 8:**
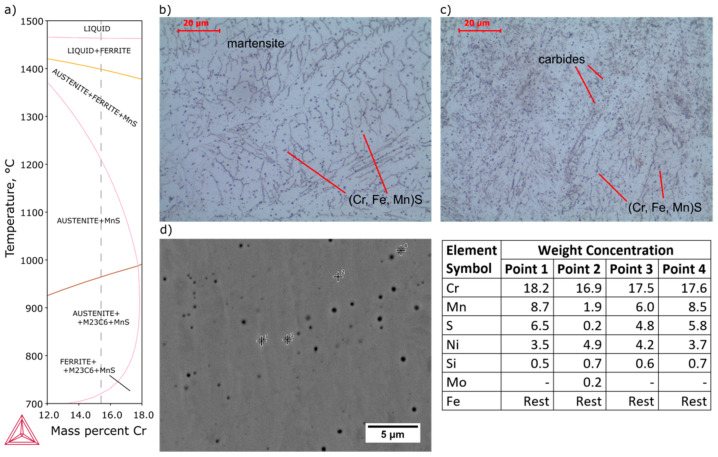
Microstructure and EDS point analysis of weld metal: (**a**) pseudobinary phase diagram of 0.11C-0.7Si-1.9Mn-0.2S-0.4Mo-4.5Ni steel, (**b**,**c**) microstructure (Kalling’s + CrO_3_ etched), (**d**) SEM microstructure and EDS points analysis (polished).

**Figure 9:**
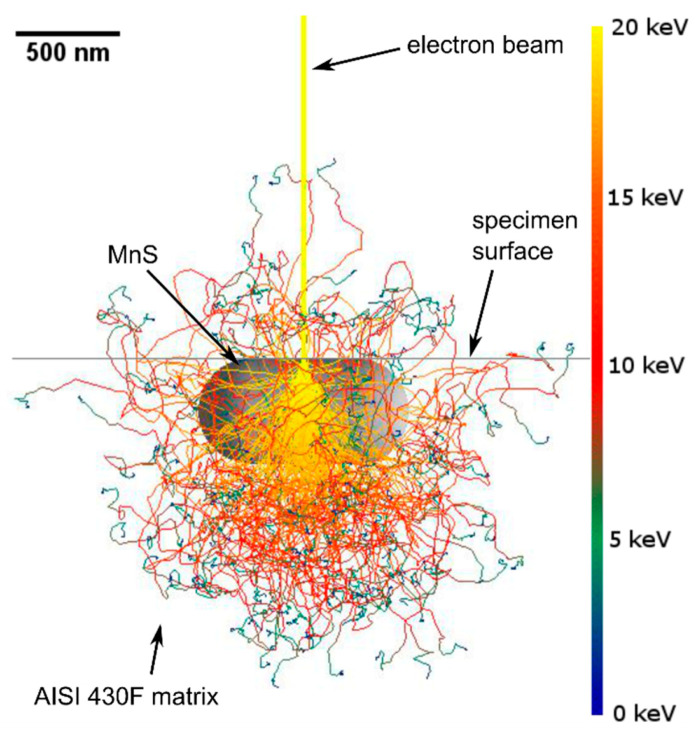
Monte Carlo Simulation of electron trajectory in MnS inclusion and AISI 430F (X12CrMoS17) matrix.

In the weld metal, martensite with many spheroidal inclusions was observed (Figure 8). δ-ferrite was not observed in microstructure, which is caused by the presence of nickel, derived from partially melted AISI 304 base metal. With increasing nickel content, the γ-field on the phase diagram expands. Accelerated cooling from homogeneous austenite transforms the austenite into martensite. EDS analysis showed enrichment of inclusions with manganese and sulfur and a similar chromium content to the matrix, which indicated that they are (Cr, Fe, Mn)S inclusions. The small size of the inclusions is responsible for the high iron content, which comes from the matrix. An example of the effective depth of electron penetration in the chemical analysis of small-size MnS inclusion, performed by Monte Carlo Simulation, is presented in Figure 9. Characteristic X-rays were emitted approximately equally from the inclusion and the matrix.

**Figure 10:**
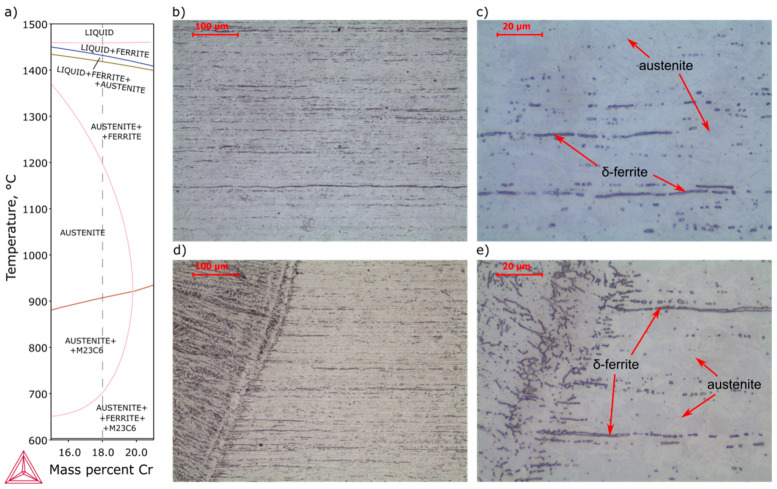
Microstructure of AISI 304 (X5CrNi18-10) steel heat-affected zone: (**a**) pseudobinary phase diagram of 0.05C-0.59Si-1.95Mn-0.009P-0.003S-0.04Mo-8.02Ni-0.05Cu steel, (**b**,**c**) base metal, (**d**,**e**) heat-affected zone (Kalling’s + CrO_3_ etched).

Base metal of AISI 304 steel (Figure 10a,b) was characterized by austenitic microstructure with banded δ-ferrite. The orientation of the δ-ferrite and a large content of twins indicate a mechanical worked delivery condition. In high-temperature HAZ (Figure 10c,d), disappearance of twins due to the recrystallization was observed, which significantly reduces strength properties [38].

**Figure 11:**
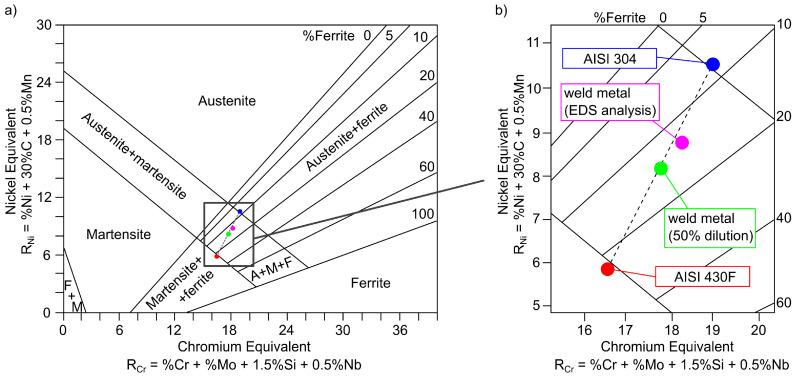
Schaeffler constitutional diagram: (**a**) general view, (**b**) enlarged view.

Comparison of the expected weld structure with the obtained results of the EDS analysis was performed with the Schaeffler diagram (Figure 11). OES analysis results of base metals, average values determined assuming of 50% dilution of both material and EDS analysis results of weld metal are presented in Table 3. In the case of AISI 430F steel, the predicted structure is martensite with approx. 24% ferrite, which essentially coincides with the obtained microstructure in the HAZ. Base metal was heat treated before welding; therefore, the structure does not correspond to that of the Schaeffler diagram. The structure of the AISI 304 steel according to the diagram is composed of austenite with approx. 8% ferrite and a small amount of martensite, which correspond to the observed microstructure. In the case of the weld, a difference between the average and measured chemical composition was identified (Table 3). Chemical composition of the weld obtained in the EDS analysis indicates that dilution of AISI 304 steel in the weld was 65% and AISI 430F was 35%. Non-axial arrangement of the laser beam in relation to the contact point of both welded materials and the difference in melting temperature and thermal conductivity coefficient values were the reasons for uneven melting. Regardless of the degree of dilution, the microstructure should consist of martensite, residual austenite and approx. 13% ferrite.

**Figure 12:**
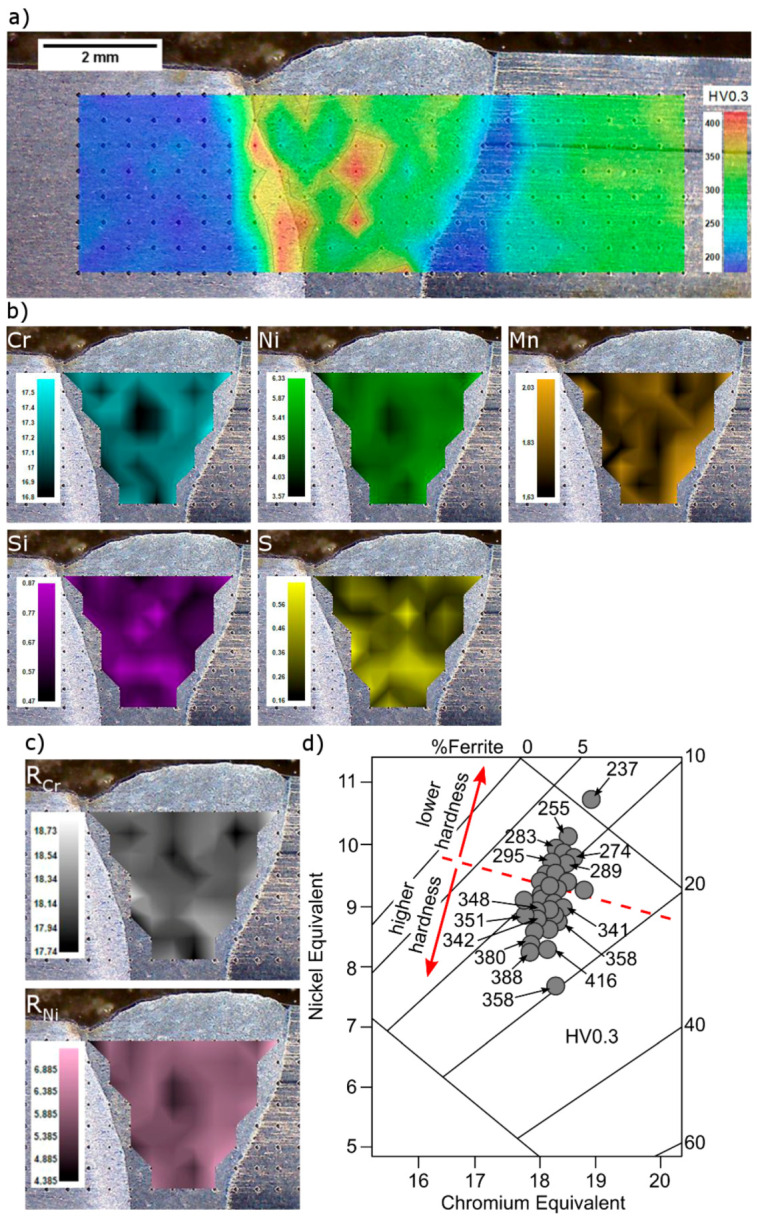
Hardness map of the joint and chemical composition distribution in weld metal. (**a**) hardness map; (**b**) chemical composition map; (**c**) chromium and nickel equivalents map; (**d**) hardness distribution depending on the chromium and nickel equivalents.

The hardness distribution map on the cross section of the joint is shown in Figure 12a. Hardness of the AISI 430F base metal was the lowest in the welded joint and amounted to 178–205 HV0.3. In the HAZ an increase in hardness to 417 HV0.3 was noted. On the AISI 304 steel side, the hardness was 259–333 HV0.3, which decreased to 191 HV0.3 in HAZ, reaching the hardness of the AISI 430F steel in the delivery condition. In the weld metal, hardness ranged from 184 to 416 HV0.3, which corresponds to the lowest and highest hardness in the other zones of the joint. The large difference in the hardness of the weld was caused by chemical segregation, shown in Figure 12b. EDS analyses were performed in the areas of 192 × 192 µm (0.037 mm^2^), where the centers of indentation and analyzed areas were a common point. The zones with the highest hardness coincide with the areas with decreased chromium and nickel contents, i.e., the main components of chromium and nickel equivalents (R_Cr_ and R_Ni_) decreased in these areas (Figure 12c). The determination of distribution of carbon in the weld metal using EDS analysis was not possible. When this distribution was simplified to be homogeneous, as the nickel equivalent value was decreased, the volume fractions of martensite and ferrite increased at the cost of austenite, and the hardness in the weld increased (Figure 12d).

## 4. Conclusions

1. Laser welded joint of AISI 430F martensitic stainless steel and AISI 304 austenitic stainless steel was characterized as C level, with reference to the ISO 13919-1 standard, due to the detection of gas pores. The final welding procedure qualification of the product has been refined on the basis of the present findings.
2. Heat input during welding and a rapid cooling cycle after welding, characteristic of laser beam welding, causes hardening of the AISI 430F steel HAZ to a mixture of martensite and ferrite, and softening of the AISI 304 steel HAZ due to recrystallization. The inclusions observed in the AISI 430F steel were Cr-rich manganese sulfides of the (Cr, Fe, Mn)S pyrrhotite type.
3. The weld metal, formed by the melting of two base metals, was characterized by the high-alloy martensite structure, in which the hardness corresponds to local changes in the chemical composition, occurring as a result of the chemical inhomogeneity of the weld. Fine, globular sulfide inclusions were also observed.

## Figures and Tables

**Table 1:** Chemical composition of base metals (OES) and weld metal (EDS).

Steel	Chemical Composition, Mass %									
	C	Si	Mn	P	S	Cr	Mo	Ni	Cu	V
AISI 430F (X12CrMoS17)	0.17	0.48	0.80	0.028	0.497	15.50	0.26	0.33	0.12	0.05
AISI 304 (X5CrNi18-10)	0.05	0.59	1.95	0.009	0.003	18.02	0.04	8.02	0.05	-
Weld metal	-	0.7	1.9	-	0.2	16.8	0.4	4.5	-	-

**Table 2:** The results of the weld and pores surface area measurements.

Zone		Area, µm^2^
**Weld Metal**		4,206,827
Pore	1	554
	2	5453
	3	5550
	4	893
	5	19,691
	6	236
	7	318
	8	80
	9	6326
	10	788
	11	3923
	12	192
	13	536
	14	1104
	15	796
	16	144
	17	3987
	18	483
	19	75
	sum	51,129

**Table 3:** Chemical composition of base metals (OES) and weld metal (arithmetic mean of the chemical composition of base metals and EDS analysis).

Steel	Chemical Composition, Mass %								
	C	Si	Mn	Cr	Mo	Ni	S	R_Cr_	R_Ni_
AISI 430F	0.17	0.48	0.80	15.50	0.26	0.33	0.497	16.48	5.83
AISI 304	0.05	0.59	1.95	18.02	0.04	8.02	0.003	18.95	10.50
Weld metal ^1^	0.11	0.54	1.38	16.76	0.15	4.18	0.25	17.72	8.17
Weld metal ^2^	0.11 ^1^	0.7	1.9	16.8	0.4	4.5	0.2	18.25	8.75

^1^ arithmetic mean of the chemical composition of base metals, ^2^ EDS analysis.
